# A double-population chaotic self-adaptive evolutionary dynamics model for the prediction of supercritical carbon dioxide solubility in polymers

**DOI:** 10.1098/rsos.211419

**Published:** 2022-01-19

**Authors:** Yan Wu, Hang Zhang, Meng-shan Li, Sheng Sheng, Jun Wang, Fu-an Wu

**Affiliations:** ^1^ School of Biotechnology, Jiangsu University of Science and Technology, Zhenjiang, Jiangsu 212018, People's Republic of China; ^2^ School of Mathematics and Computer Science, Gannan Normal University, Ganzhou Jiangxi 341000, People's Republic of China; ^3^ College of Physics and Electronic Information, Gannan Normal University, Ganzhou Jiangxi 341000, People's Republic of China; ^4^ Sericultural Research Institute, Chinese Academy of Agricultural Sciences, Zhenjiang, Jiangsu 212018, People's Republic of China

**Keywords:** dissolution behaviour, evolutionary computation, particle dynamics, computational model

## Abstract

Solubility of gas in polymers is an important physico-chemical property of foam materials and widely used in the preparation and modification of new materials. Under the conditions of high temperature and high pressure, the dissolution process is a nonlinear, non-equilibrium and dynamic process, so it is difficult to establish an accurate solubility calculation model. Inspired by particle dynamics and evolutionary algorithm, this paper proposes a hybrid model based on chaotic self-adaptive particle dynamics evolutionary algorithm (CSA-PD-EA), which can use the iterative process of particles in evolutionary algorithms at the dynamic level to simulate the mutual diffusion process of molecules during dissolution. The predicted solubility of supercritical CO_2_ in poly(d,l-lactide-*co*-glycolide), poly(l-lactide) and poly(vinyl acetate) indicated that the comprehensive prediction performance of the CSA-PD-EA model was high. The calculation error and correlation coefficient were, respectively, 0.3842 and 0.9187. The CSA-PD-EA model showed prominent advantages in accuracy, efficiency and correlation over other computational models, and its calculation time was 4.144–15.012% of that of other dynamic models. The CSA-PD-EA model has wide application prospects in the computation of physical and chemical properties and can provide the basis for the theoretical calculation of multi-scale complex systems in chemistry, materials, biology and physics.

## 1. Introduction

Solubility of supercritical CO_2_ in polymers is the key factor affecting the performance of foamed materials and also widely used in the extraction and separation of various chemicals and the preparation and modification of new materials, displaying good prospects in theoretical research and application [1–6]. Under the supercritical conditions of high temperature and high pressure, the dissolution behaviour is affected by many factors and shows nonlinear, non-equilibrium, dynamic and critical properties, which result in the difficulty in the study of dissolution experiments [7–15]. The current information technology and artificial intelligence have provided theoretical and technical support for the calculation and simulation of material properties. The calculation of dissolution behaviour is important in theoretical studies and applications.

In recent years, dissolution calculation models have been widely considered and mainly include traditional models based on mathematical theory and computational models based on information technology [12,16–22]. Mathematical models mainly include thermodynamic equation of state models and empirical/semi-empirical models. The equation of states is mainly Peng–Robinson and Sanchez–Lacombe equations of state and empirical models [23–25]. Computational models mainly include *ab initio* method, molecular simulation and artificial intelligence method [26,27]. The *ab initio* method is a quantum mechanics calculation method and can afford information of microstructures and molecular interaction force by solving the Schrödinger equation. Molecular simulation is a calculation technology based on classic Newton mechanics and can be classified into Monte Carlo simulation and molecular dynamics simulation [28–31]. The artificial intelligence method can be used to establish computational model based on artificial intelligence algorithms, such as neural network and intelligent algorithm, for predicting dissolution behaviours. Compared with mathematical models, the dissolution calculation model based on artificial neural network was better, but the performance of the artificial neural network model was excessively dependent on its training algorithm [32–35]. Particle swarm optimization (PSO) was used to predict the phase equilibrium data of supercritical CO_2_, and good prediction results were obtained [36]. When the solution was close to the global optimal solution, the search process of single PSO was slow, and a local optimal solution might be obtained [37]. The combined algorithm of PSO and back propagation (BP) algorithms showed better performance than a single algorithm [38]. Our team had also carried out related studies, including but not limited to the group evolution algorithm combined with the BP algorithm [39], clustering and diffusion theory and proposed several solubility prediction models of supercritical CO_2_ in polymers under several macroscopic scales in order to improve the local search speed [40,41]. These models achieved better results in calculation accuracy, efficiency, correlation and comprehensive performance.

The above models have improved calculation accuracy and efficiency, but there are still some problems to be further solved. Firstly, the solubility models based on artificial intelligence have some obvious shortcomings, such as over-reliance on the explored samples and the training data. Secondly, the models do not consider the essence of dissolution or analyse the reasons for dissolution or the mechanism of dissolution. Thirdly, although molecular simulation reveals the dissolution properties of the system from various points of view, the simulation process considers various fields of forces and is characterized by long computation time and low calculation accuracy.

The dissolution process is essentially the mutual diffusion of molecules, the solvent–solute mass transfer process. Under the action of potential force field in the system, solute molecules gradually diffuse into a solvent and finally reach dissolution equilibrium. The diffusion trajectory of molecules during dissolution can be simulated with particle motion in the dynamic potential energy field in the swarm intelligence algorithm. The computational process of particle dynamics and evolution algorithms is actually an evolutionary process of the property of discrete particles in a series of time intervals. Inspired by the essence of dissolution, this paper combines the swarm intelligence algorithm with particle dynamics to allow particles in the evolution algorithm to iterate in the dynamic potential field and proposes a mixed prediction model based on bi-group evolution strategy algorithm and particle dynamic method. Through the computational experiments of dissolution behaviour of supercritical carbon dioxide in poly(d,l-lactide-*co*-glycolide) (PLGA), poly(l-lactide) (PLLA) and poly(vinyl acetate) (PVAc), the computational performance of the mixed model is analysed.

The innovation and contribution of this paper are summarized as follows. (i) Through combining particle dynamics with evolutionary algorithms, a mixed model is proposed. (ii) In the model, the swarm evolution algorithm replaces various integral algorithms in traditional dynamics. (iii) This model has broad application prospects in multi-scale and dynamics calculation.

## 2. Model theory

### 2.1. Chaotic self-adaptive evolutionary algorithm

The PSO algorithm is one of the classic swarm intelligence algorithms. Through simulating the food search process of a bird population, PSO can search for the optimal solution, and its advantages include fewer parameters and fast optimization [42,43].

When solving problems with PSO, each possible solution is set as a particle of a population. Each particle flies at a regular speed in the solution space, and each flight produces a new set of feasible solutions. If there are *m* particles in the *n*-dimensional search space, $X={\left(x_{1,}x_{2,}\ldots ,x_{n}\right)}^{T}$ is particle population and $x_{i}={\left(x_{i,1,}x_{i,2,}\ldots ,x_{i,n}\right)}^{T}$ and $v_{i}={\left(v_{i,1,}v_{i,2,}\ldots ,v_{i,n}\right)}^{T}$are respectively the position and speed of the *i*th particle. The particle evolution formula is provided as follows:

2.1 $$v_{i,d}^{k+1}=\omega v_{i,d}^{k}+c_{1}(p_{i,d}^{k}-x_{i,d}^{k})+c_{2}(p_{g,d}^{k}-x_{g,d}^{k})$$

and

2.2 $$x_{i,d}^{k+1}=x_{i,d}^{k}+v_{i,d}^{k+1},$$

where $v_{i,d}^{k}$, $x_{i,d}^{k}$ and $p_{i,d}^{k}$ are respectively the speed, position and individual value of the *i*th particle in the *d*th dimension's iteration; $p_{g,d}^{k}$ is the global extreme value of the population; ω is called inertial weight factor; $c_{1}$ and $c_{2}$ are respectively developmental learning factor and exploratory learning factor.

Inertial weight factor, developmental learning factor and exploratory learning factor are three important parameters affecting algorithm performance and determine respectively the inertia of particles, search capability and exploration capability. A larger inertial weight factor is beneficial to the global extreme value, whereas a smaller one is beneficial to the local extreme value. A larger inertial coefficient is beneficial to global search, whereas a smaller inertial coefficient is beneficial to local search [44–46]. Cognitive learning factor and exploratory learning factor are collectively called learning factor, and appropriate adjustment of learning factors can balance global search efficiency and local search efficiency, thus improving convergence speed and accuracy and improving diversity. Therefore, this paper used a chaos theory and adaptive strategy to adjust the parameters of the algorithm and obtained the chaotic self-adaptation PSO evolution algorithm. In the algorithm, the learning factors $c_{1}$ and $c_{2}$ are produced by the Lorenz equations as follows:

2.3 $$\left\{ \begin{array}{l} \frac{dx}{dt}=-a(x-y),\;\\ \frac{dy}{dt}=rx-y-xz,\;\\ \frac{dz}{dt}=xy-bz.\; \end{array}\right.$$

In equation (2.3), if the values of *a*, *b* and *r* are respectively 10, 8/3 and 28, the system is in a chaotic state. Therefore, learning factors (*c*_1_ and *c_2_*) are defined as follows:

2.4 $$\left\{ \begin{array}{c} c_{1}=x(t),\;\\ c_{2}=y(t).\; \end{array}\right.$$

The inertial weight factor adjustment formula is provided as follows:

2.5 $$\omega =\omega_{\max}-\mathrm{Pgbest}(k)/\mathrm{Plbes}t_{\mathrm{ave}}-(\omega_{\max}-\omega_{\min})\;\times k/k_{\max},$$

where *ω*_max_ and *ω*_min_ are respectively expressed as the maximum and minimum inertia weights; Pgbest (*k*) is the global extreme value of the *k*th iteration; Plbest_ave_ is a local average value; *k*_max_ is the maximum number of iterations; *k* is the current iterative number.

### 2.2. Particle dynamics evolutionary algorithm

Particle dynamics refers to statistics of physical, chemical and mechanical properties of a system based on the motion state of each particle. In particle dynamics, the motion equation is established through the potential capacity field to calculate digital integrals of the equation and obtain relevant properties and parameters. The calculation process of particle dynamics is the evolution process of particles within a period of time, mainly three steps, including particle initialization, calculation of particle potential energy and statistics of particle properties [47,48]. It can be seen from the potential energy field that the force on particle *q* is composed of three forces: conservative force (*F_C_*), dissipative force (*F_D_*) and random force (*F_R_*):

2.6 $$F_{q}=\sum F_{C}^{q}+F_{D}^{q}+F_{R}^{q}.$$

The total potential energy of particles is expressed as follows:

2.7 $$u(r_{q})=\sum_{\;j=1,j\neq q}^{N}u_{qj}(r_{q},r_{j}).$$

Thus, the force is

2.8 $$F_{q}=-\nabla u(r_{q}).$$

The acceleration is

2.9 $$a_{q}=\frac{F_{q}}{m}=\frac{-\nabla u(r_{q})}{m},$$

where *m* is the mass of the particle. According to Newton's law, with acceleration, the speed and position of the particles can be calculated, and the evolution of the system over time can be obtained by repeated iterations.

In this paper, the above evolution algorithm is combined with particle dynamics to obtain chaotic self-adaptive particle dynamics evolutionary algorithm (CSA-PD-EA). In CSA-PD-EA, dynamic particles are coarsely classified according to certain rules and the evolution particles are obtained after classification. Evolutionary particles move in the dynamic level, so that evolutionary particles have dynamic potential energy. Based on the dynamics field theory, the total potential energy of the evolutionary particles is calculated to obtain the role, acceleration, speed and position. Finally, the number and mass of particles in the statistical region are counted based on the locations of particles and the mass of particles is the amount of dissolved particles.

In a CSA-PD-EA model involving *R* moving particles, in the Lennard-Jones potential capacity field, the total potential energy is

2.10 $$\left\{ \begin{array}{l} \begin{array}{cc} E(u_{q})=\sum_{q=1}^{R}\sum_{\;j=1,j\neq q}^{R}4\varepsilon \left[{\left(\frac{\delta }{r_{qj}}\right)}^{12}-{\left(\frac{\delta }{r_{qj}}\right)}^{6}\right] & r_{qj}\leq r_{c}, \end{array}\;\\ \begin{array}{cc} E(u_{q})=0 & r_{qj}>r_{c}, \end{array}\; \end{array}\right.$$

where $E(u_{q})$ is the potential energy of the particle *q*; $r_{qj}$ is the distance between particles *q* and *j*; ε and δ are respectively the energy and distance parameters; $r_{c}$ is the cutoff.

In the CSA-PD-EA model, the speed and location are respectively updated as

2.11 $$\left\{ \begin{array}{l} x_{q}^{k+1}=x_{q}^{k}+c_{2}v_{q}^{k+1}+c_{1}r,\;\\ v_{q}^{k+1}=\omega a_{q}^{k},\;\\ a_{q}^{k}=-\frac{\nabla E(u_{q}^{k})}{Rm}.\; \end{array}\right.$$

## 3. Model establishment

**Figure 1.**
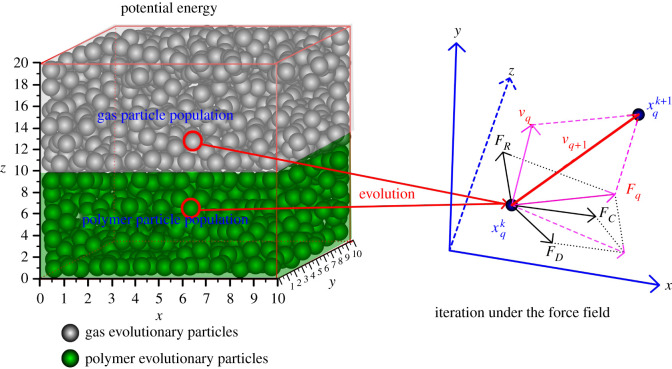
Iteration direction of evolutionary particles.

The CSA-PD-EA model was built in a cube of 10 × 10 × 20 (figure 1). Evolutionary particles are divided into two layers. The upper layer is supercritical gas evolutionary particles, and the lower half is polymer evolutionary particles. The junction between the two layers is the interface layer. Supercritical carbon dioxide particles and polymer particles diffuse into each other through the interface layer, thus eventually achieving dynamic equilibrium and saturation.

In the model, two populations are set according to the evolutionary particle species: supercritical carbon dioxide particles and polymer particle populations. Through the potential force field between populations, the particles of the two populations diffuse to each other and finally reach the equilibrium state. In an equilibrium state, the motion of evolutionary particles between populations is dynamically balanced, indicating that the mutual movement of supercritical carbon dioxide particles and polymer particles is dynamically balanced.

**Figure 2.**
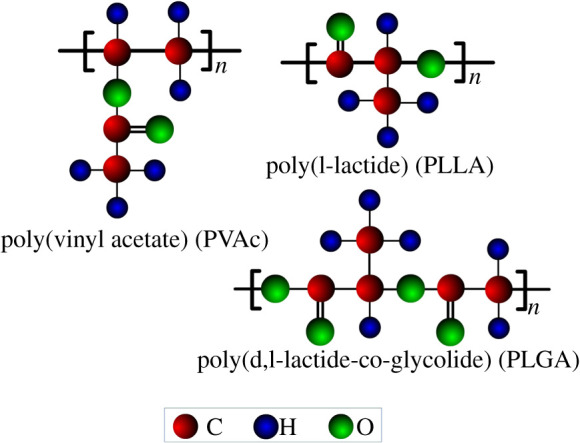
Classification of polymer evolutionary particles.

The CSA-PD-EA model consists of evolutionary gas particles and evolutionary polymer particles. In order to bring the molecular mass of each evolutionary particle closer together, every 100 CO_2_ molecules form one CO_2_ evolutionary particle. Each polymer is divided into evolutionary particles according to the components, as shown in figure 2. Each evolutionary polymer particle contains 34 PLGA, 52 PVAc and 62 PLLA molecules.

**Figure 3.**
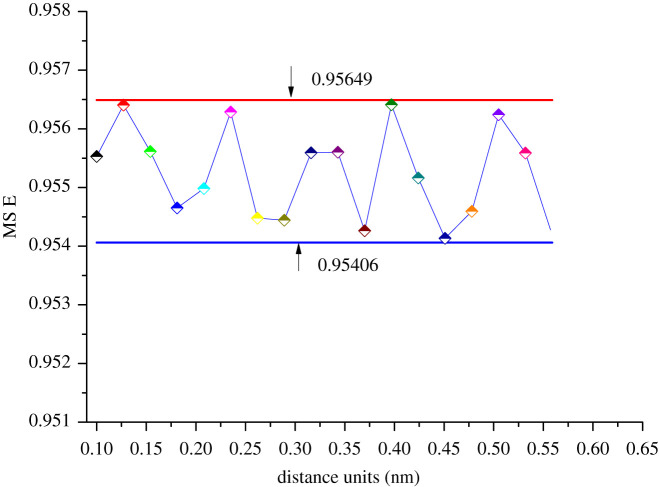
Variations of MSE with distance unit *ε* = 2.893.

**Figure 4.**
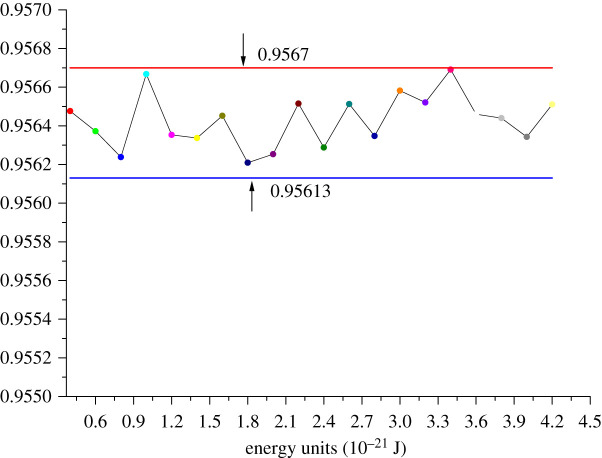
Variations of MSE with energy unit *δ* = 0.2378.

The CSA-PD-EA model has three kinds of polymer particles and one kind of gas particles. In total, seven pairs of evolutionary particles are respectively SCCO_2_–SCCO_2_, SCCO_2_–PLLA, SCCO_2_–PLGA, SCCO_2_–PVAc, PLLA–PLLA, PLGA–PLGA and PVAC–PVAC. In order to analyse the effect of each potential energy parameter on the performance of the model, modelling experiments were performed under different conditions of various parameters. Taking PLLA–PLLA as an example, figures 3 and 4 show the calculation error curves of the model under different potential energy parameters.

The MSE curve remains stable when distance units and energy units change, and the data are distributed within a small interval with an interval error width of no more than 0.002. The computational performance of the CSA-PD-EA model is not absolutely correlated with potential energy parameters. The average of each parameter is calculated. Electronic supplementary material S2 gives potential energy parameters which are used in modelling. Since this paper focuses on the calculation of the solubility of SCCO_2_ in polymers, the number of polymer particles diffused to the SCCO_2_ particle population is no longer counted.

## 4. Results and discussion

The CSA-PD-EA model was built in Windows 7 SP1 64-bit OS (4.00 GB of memory and Intel (R) Core i5-4460 processor) and programmed and calculated with Matlab 2010a. The experimental datasets, parameters, operating environment and code supporting this article have been uploaded as part of the electronic supplementary material. The coordinate position was recorded by tracking the motion trajectory of each evolutionary particle. This paper explored the evolution process of supercritical carbon dioxide particles and the density and radial distribution function (RDF) of polymer population and then analysed the solubility of supercritical carbon dioxide particles in polymers. Finally, the comprehensive performance of the proposed model was compared with that of other models.

### 4.1. Evolution of gas evolution particles over time

**Figure 5.**
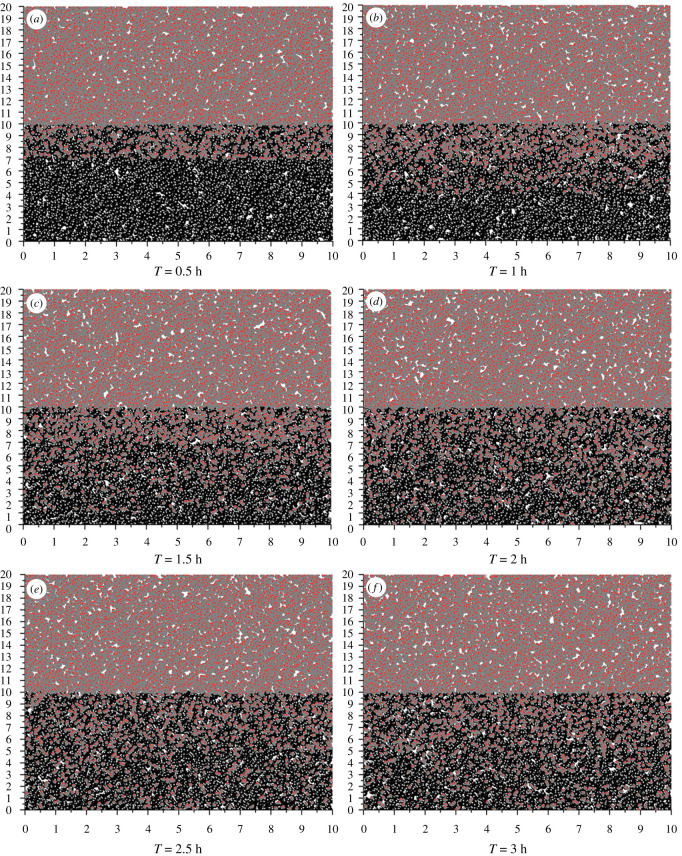
Snapshots of SCCO_2_ particles dissolved in PLLA at different times.

After the model is initialized, the two-phase evolution particles are distributed over the upper and lower layers. In the iterative updating process of evolutionary particles, the two-phase particles diffuse to each other. After a period of iteration, the dissolution process is balanced finally. This paper only discusses the diffusion of carbon dioxide particles into the polymer particle melt. Taking PLLA as an example, figure 5 shows the variations of the positions of supercritical carbon dioxide evolutionary particles in PLLA particles with time.

**Figure 6.**
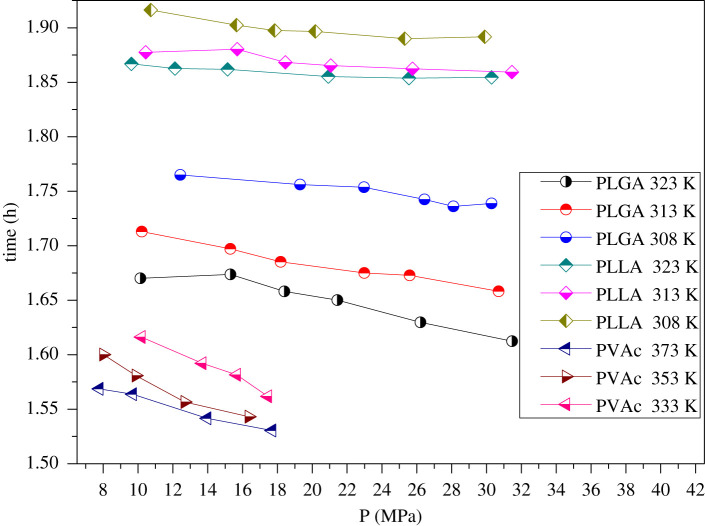
Equilibrium evolution time under different pressures and temperatures.

After half an hour, a small number of gas particles were distributed in the polymer melt below the interface, indicating that some gas particles are dissolved in the polymer melt. After 1 and 1.5 h of evolution, gas particles were increasingly dense in the vertical direction of the polymer melt, indicating that the particles dissolved in the polymer were gradually incremented. After 2 h, the distribution of evolutionary gas particles remained dynamic, indicating the dissolution balance of the system was achieved after 2 h. At the balance dissolution point, the number of gas particles in the polymer melt was counted, and the solubility was calculated. The evolution of gas particles in PLGA and PVAc polymers is similar to that in PLLA, so the evolution of gas particles in PLGA or PVAc polymers is not discussed in this paper. Figure 6 shows the curves of dynamic equilibrium time of each polymer with temperature and pressure.

Under the conditions of the same polymer and the same temperature, equilibrium time decreased gradually with the increase in pressure. Equilibrium time decreases with the increase in pressure and temperature. The dissolution equilibrium time of supercritical carbon dioxide in different polymers was slightly different, but it was in the range of 1.5–2.0 h.

### 4.2. Density of gas particles in polymer particles

**Figure 7.**
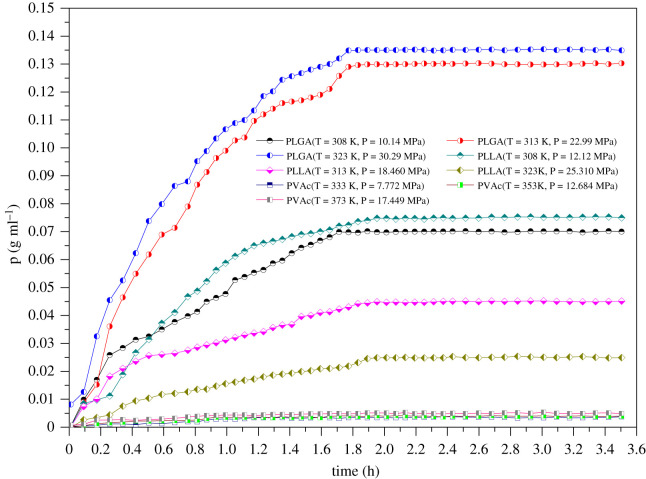
Density evolution curve of SCCO_2_ in polymer.

After the evolution process of particles, gas particles slowly diffuse into polymer particles, and the density of gas particles in the polymer gradually increases and finally achieves the equilibrium state. In order to quantify the number of carbon dioxide particles in the polymer, the density curve of carbon dioxide particles in each polymer was plotted (figure 7).

**Figure 8.**
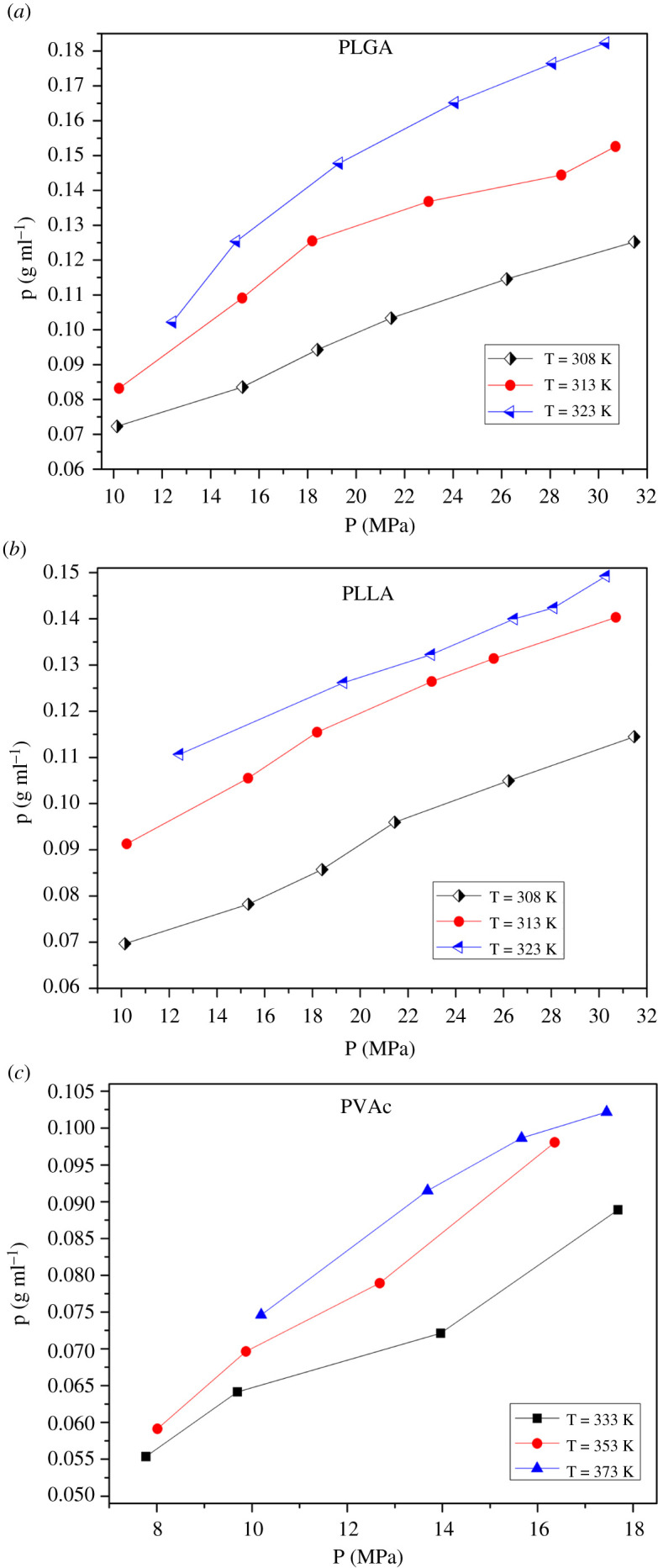
Variation of SCCO_2_ density with pressure and temperature in each polymer. (*a*) PLGA, (*b*) PLLA and (*c*) PVAc.

The density of gas particles firstly increased quickly, then rose slowly and finally achieved the equilibrium state, indicating the dissolution rate of gas particles in polymers was first fast, then became slow, and finally reached the dynamic equilibrium state. The density equilibrium time was between 1.0 and 1.4 h. The equilibrium time of carbon dioxide particles in PVAc, PLLA and PLGA gradually increased, and the density equilibrium time also increased gradually. In order to explore the relationship between the density and temperature of gas particles in the polymer in the equilibrium state, the density equilibrium curves of gas particles in each polymer at different temperatures were plotted (figure 8).

In the same polymer, at the same temperature, the gas density increases with the increase in the system pressure. The gas density shows a nonlinear proportional relationship with pressure. Under the same pressure, the density decreases with the increase in temperature, indicating that the gas density shows a nonlinear inverse relationship with the system pressure.

### 4.3. Radial distribution functions of evolutionary gas particles

**Figure 9.**
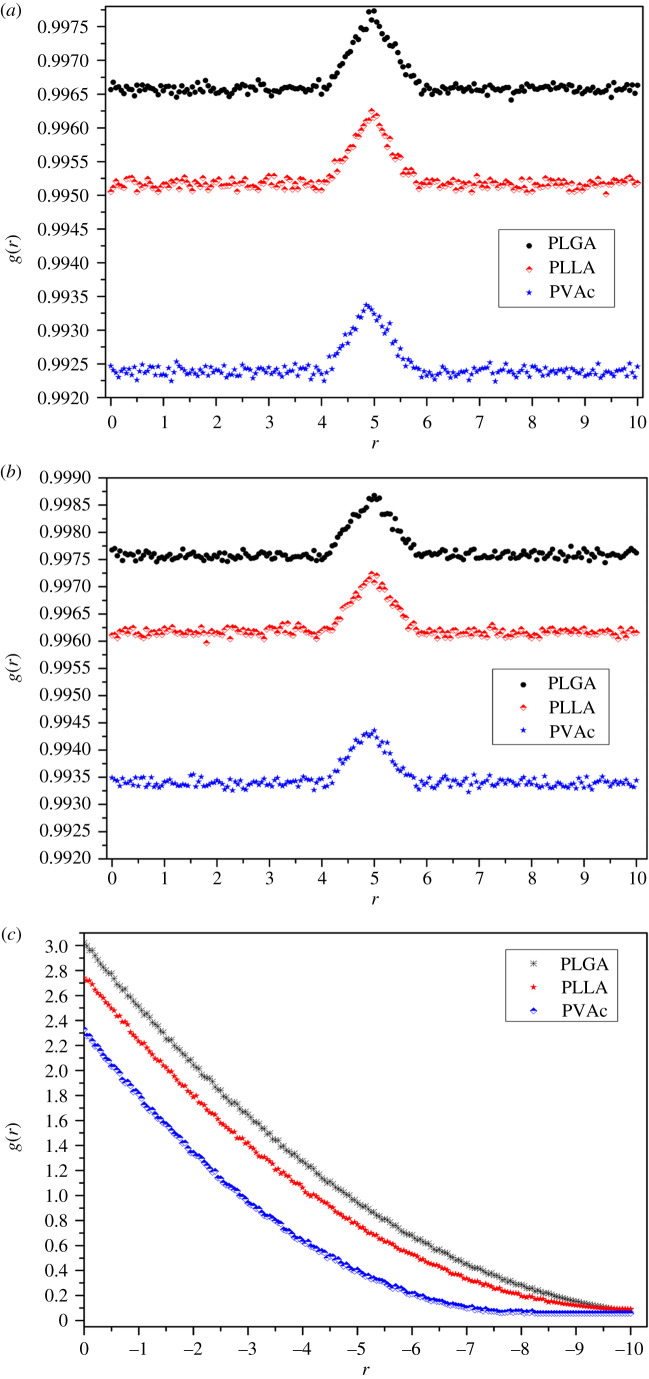
RDF curves of SCCO_2_ particles in three polymers: (*a*) *x*-axis direction, (*b*) *y*-axis direction and (*c*) *z*-axis direction. PLGA (*T* = 313.00 K, *P* = 22.990 MPa), PLLA (*T* = 313.00 K, *P* = 18.460 MPa), PVAc (*T* = 373.00 K, *P* = 15.664 MPa).

In order to display the distribution of particles in the calculation space intuitively, the RDF was used to explore the distribution of carbon dioxide particles in the polymer melt in this paper. In the cube of 10 × 10 × 20, the centre of a vertical side in the two-phase interface is taken as the origin of coordinates (also a reference point of RDF) to establish the three-dimensional coordinate system, and then a rectangular box with a height in the vertical direction is established based on a plane of the three-dimensional coordinate system. The RDF is defined as the ratio *g*(*r*) of the gas particle region density and the global particle density, and the region density is the ratio of the carbon dioxide particles to the cube volume. The global density is the ratio of total particle number of polymer melt to polymer melt volume. Figure 9 shows the RDF curves of SCCO_2_ particles in each polymer in the *x*, *y* and *z* directions.

The trends of the RDF curves of gas particles in the *x*, *y* and *z* directions are basically the same. In both *x* and *y* directions, the RDF curves are basically stable except that a small crest appears near the no. 5 coordinate position, indicating that carbon dioxide particles are distributed uniformly in the *x* and *y* directions. The crest indicates that the distribution density at the central point is slightly smaller than that at other locations. The difference between the maximum and minimum RDF values in the curve crest is less than 0.001. In the *z* direction, the RDF curve gradually descends from the reference point and finally becomes stable. The RDF curve in the *z* direction reflects the distribution characteristics of particles in the vertical direction. The density of carbon dioxide particles in the vertical direction gradually becomes smaller. Near the reference point, the particle density is high, indicating that SCCO_2_ particles are densely distributed at the interface layer. The lower the position is, the smaller the particle density is, indicating that there are fewer particles. Finally, at the bottom of the cube, the particles are sparser, and the density is smaller.

Starting from the two-phase interface layer, SCCO_2_ particles gradually vertically diffuse to the bottom layer of the polymer, and the diffusion density is getting smaller. In the horizontal direction, the particle distribution is basically uniform. In the same vertical height, the distribution of SCCO_2_ particles is also basically uniform. At the centre of the cube, the SCCO_2_ particle density is slightly higher than that at other positions.

### 4.4. Comparative analysis

In order to further verify the computation capacity and advantages of the CSA-PD-EA model, this paper compares the calculation values of the model and previous experimental values [49–54]. The performances of the proposed model and other predictive models are compared (table 1).

**Table 1.** Comparison models.

model	model details	references
MD	molecular dynamics	[55]
AIMD	*ab initio* molecular dynamics	[56]
SDPD	smooth dissipative particle dynamics	[57]
CSPSO BP ANN	method based on chaos theory, self-adaptive particle swarm optimization (PSO) algorithm and back propagation artificial neural network	[58]
DP-DT-CAPSO RBF ANN	model based on the diffusion theory and an improved double-population chaotic accelerated particle swarm optimization (CAPSO) algorithm	[59]

In Matlab 2010a, the above model (for the parameters of the model, refer to the corresponding document) was programmed, and the calculation experiment of SCCO_2_ dissolution in the polymer was performed. The calculation accuracy of each model was assessed with average relative deviation (ARD) and mean square error (MSE), and model correlation was evaluated with squared correlation coefficient (*R*^2^) as follows:

4.1 $$\mathrm{ARD}=\frac{1}{N}\sum_{i=1}^{N}\frac{\left|{\bar{y}}_{i}-y_{i}\right|}{y_{i}},$$

4.2 $$\mathrm{MSE}=\sqrt{\frac{1}{N}\sum_{i=1}^{N}{(y_{i}-{\bar{y}}_{i})}^{2}}$$

4.3 $$\;\mathrm{and}\;R^{2}=\frac{{\left[\sum_{i=1}^{N}(y_{i}-y_{\mathrm{ave}})({\bar{y}}_{i}-{\bar{y}}_{\mathrm{ave}})\right]}^{2}}{\sum_{i=1}^{N}{(y_{i}-y_{\mathrm{ave}})}^{2}\sum_{i=1}^{N}{({\bar{y}}_{i}-{\bar{y}}_{\mathrm{ave}})}^{2}},$$

where *N* is the sample number; ${\bar{y}}_{i}$ and ${\bar{y}}_{\mathrm{ave}}$ are respectively the predicted value and the average predicted value; $y_{i}$ and $y_{\mathrm{ave}}$ are respectively the experimental value and the average experimental value.

#### 4.4.1. Dissolution result after balance

**Figure 10.**
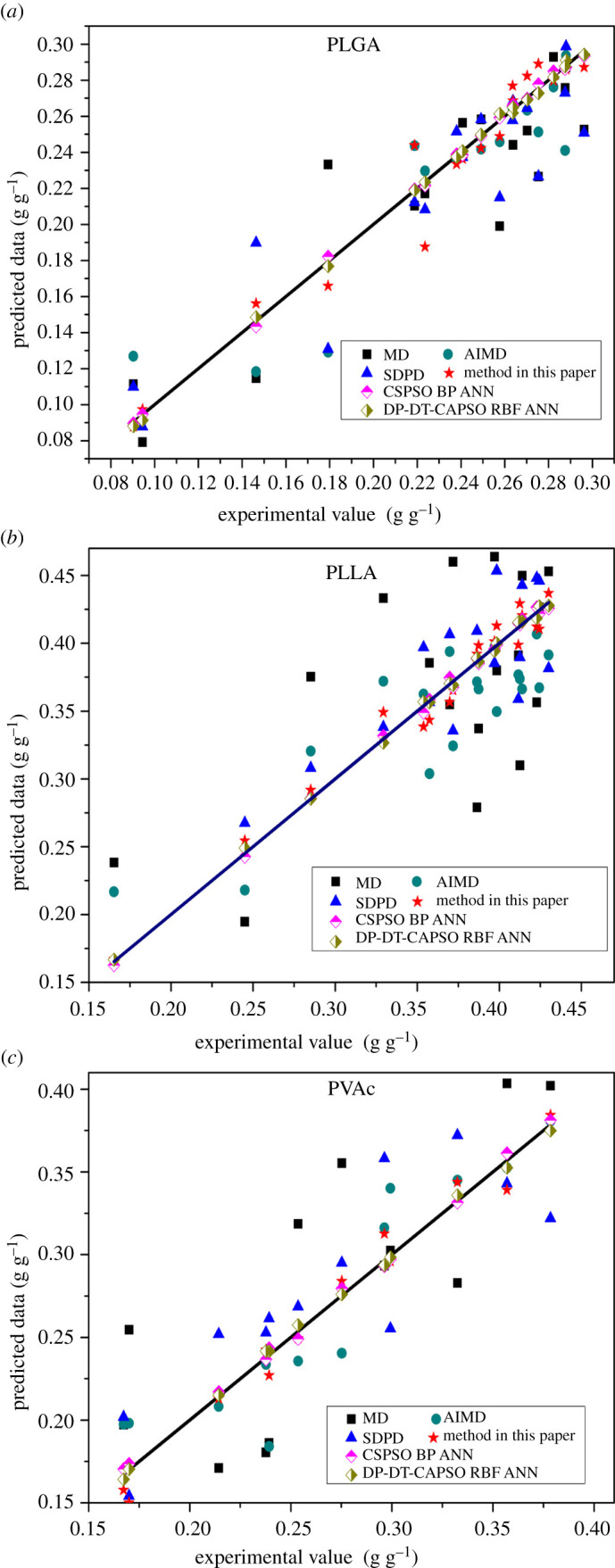
Comparison between experimental values and predicted values obtained with various models: (*a*) PLGA (*T* = 308 K, 313 K, 323 K, *P* = 10.220–31.470 MPa), (*b*) PLLA (*T* = 308 K, 313 K, 323 K, *P* = 9.620–31.460 MPa) and (*c*) PVAc (*T* = 333 K, 353 K, 373 K, *P* = 7.772–17.685 MPa).

The solubility of gas particles in the polymer under different pressures and temperatures was obtained by the solubility calculation experiment of each model. Figure 10 shows the calculated and experimental values of the solubility obtained with different models.

The straight line is an ideal state line indicating that the model calculation values are equal to experimental values. The data points indicate the calculation values of each model. The vertical distance between the calculated data points and the ideal line indicates the error between the predicted value and the experimental value and reflects the calculation accuracy of the models. The larger the vertical distance is, the greater the prediction error is. In three polymer experiments, the data point distribution of the MD method is far from the straight line. The data point distributions of AIMD and SDPD are substantially the same. The calculated data points of the CSA-PD-EA model proposed in this paper are closer to the straight line. The calculated data points of CSPSO BP ANN and DP-DT-CAPSORBFANN models are near to the straight line. The calculation error of the MD method is large, whereas the calculation errors of AIMD and SDPD models are smaller. The calculated values of the CSA-PD-EA model proposed in this paper are closer to the experimental value, and its error is small. The calculated values of both CSPSO BP ANN and DP-DT-CAPSO RBF ANN models are close to the straight line, indicating small errors and high prediction accuracy.

The prediction accuracy of the model based on artificial neural network is obviously better than that of the three models based on particle dynamics. Since the calculation of the model based on the artificial neural network is essentially data analysis and processing at the macro level, the model can dynamically adjust the error between input and output by changing internal parameters so as to improve the prediction accuracy. Although the four models based on particle dynamics are not as accurate as the artificial neural network model in macroscopic calculation, they have a more significant mechanism interpretability and can simulate the dissolution process of particles. Table 2 shows the related performance indicators of each model. The calculation time refers to the period from the beginning of the model calculation to the dissolution equilibrium time.

**Table 2.** Performance evaluation index of each model.

model	ARD	MSE	*R*^2^	CPU time (s)
MD	2.3452	1.5263	0.7626	158 679.50
AIMD	1.7798	1.3126	0.7896	110 023.56
SDPD	1.5963	0.9599	0.8213	45 523.44
method in this paper	1.0206	0.3842	0.9187	6798.58
CSPSO BP ANN	0.1275	0.0120	0.9966	10.89
DP-DT-CAPSO RBF ANN	0.0033	0.0114	0.9973	25.77

CSPSO BP ANN and DP-DT-CAPSO RBF ANN models have obvious advantages in calculation accuracy and correlation (table 2). The accuracy and correlation of AIMD are close to those of SDPD, and the accuracy and correlation of the SDPD model are slightly better. Compared to MD, AIMD and SDPD, the CSA-PD-EA model proposed in this paper has better accuracy and higher correlation. In the computation process, CSPSO BP ANN and DP-DT-CAPSO RBF ANN models only involve training and test and require less time, but the data analysis models are only better at the macro level. Calculation time of dynamic models decreases according to the following order: MD > AIMD > SDPD > CSA-PD-EA. The computational efficiency of the SA-PD-EA model is nearly 20 times higher than that of the MD model.

#### 4.4.2. Changes of particle number

**Figure 11.**
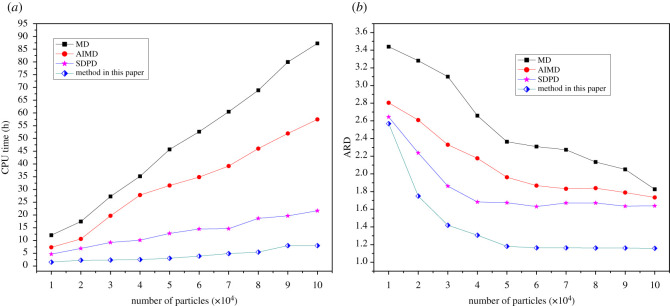
Variations of calculation time and accuracy with particle size.

In order to compare the computational results of each dynamic model under different numbers of particles, the computational time and accuracy of each model after 100 000 iterations are plotted in figure 11.

As can be seen from figure 11*a*, calculation time of each model increases with the increase in the number of particles, indicating that calculation time is proportional to particle number. The degree of tilt of each time curve reflects the variation of calculation time with particle number. Calculation time of the MD model increases greatly with the increase in particle number, whereas that of the CSA-PD-EA model is relatively stable, indicating that calculation time of this model does not increase rapidly with the increase in particle number. The accuracy curve of each model decreases with the increase in particle number (figure 11*b*), indicating that the calculation error decreases with the increase in particle number. After the number of particles increases to 50 000, the calculation error of the CSA-PD-EA model basically remains unchanged, indicating that the calculation accuracy cannot be improved by adding evolved particles when the quantity of evolutionary particles reaches a certain value. The results show that increasing the number of particles also increases the calculation cost and improves the calculation accuracy, but the calculation accuracy is seldom changed when the number of particles reaches a certain value.

#### 4.4.3. Comparative analysis and discussion

(i) CSPSO BP ANN and DP-DT-CAPSO RBF ANN have obvious advantages in the calculation accuracy, correlation and calculation efficiency in the solution prediction and show good comprehensive performance. However, the dissolution velocity cannot be calculated with CSPSO BP ANN or DP-DT-CAPSO RBF ANN or reflect the dissolution change of particles in the system with time. CSPSO BP ANN and DP-DT-CAPSO RBF ANN largely rely on experimental data. With experimental data, the models cannot be trained for prediction. In addition, the models cannot provide theoretical support for the mechanism of dissolution.
(ii) The four prediction models based on dynamics (MD, AIMD, SDPD and CSA-PD-EA models) can fully calculate the state change of each particle with time and the dissolution rate. The models do not depend on experimental data and can be used for dissolution calculation without experimental data, thus overcoming the shortcomings of data-based fitting prediction models. In addition, the temperature and pressure of the model can be set arbitrarily without any restrictions on temperature and pressure of the system, which is conducive to the expansion of the model. Moreover, the calculation efficiency of the CSA-PD-EA model is 5–20 times higher than that of similar dynamics models. The CSA-PD-EA model has small calculation error and significantly improved correlation and calculation accuracy.
(iii) In order to confirm the performance of the CSA-PD-SA model in different polymer systems, the parameters of each polymer are shown in table 3. The data show that the performance of the proposed model in the three polymers is slightly different. Whether it is ARD, MSE, *R*^2^ or CPU time, the overall performance of all polymer systems is at the same level, and PLLA is slightly dominant.
(iv) In the dissolution interpretability, the CSA-PD-EA model has a unique advantage. The essence of dissolution is the physical change of the mutual fusion, a solute diffusion process into solution. In the gas–polymer system, gas particles are diffused into the polymer melt and finally achieve dissolution equilibrium. In the CSA-PD-EA model, the particle evolution process is used to simulate the diffusion process of solute into the melt. Therefore, the CSA-PD-EA model has significantly improved comprehensive performance in calculation error, correlation, and calculation accuracy.

**Table 3.** Performance evaluation parameters of each polymer in the CSA-PD-EA model.

polymer	ARD	MSE	*R*^2^	CPU time (s)
PLGA	1.0293	0.3900	0.91586	6974.57
PLLA	1.0101	0.3779	0.92306	6584.59
PVAc	1.0224	0.3847	0.91716	6836.58
average value	1.0206	0.3842	0.9187	6798.58

Due to the interpretability of the model, although this paper only shows the test performance in three polymers, it is reasonable to conclude that for other polymers it will also perform well. At the same time, it can also be used for reference in other molecular systems. Due to the limitation of space and the different emphasis of the article, this paper does not expand and discuss them in detail.

## 5. Conclusion and outlook

In this paper, a hybrid model based on chaotic self-adaptive PSO and particle dynamics, the CSA-PD-EA model, is proposed by integrating population evolution algorithm with particle dynamics, and the performance of the model is demonstrated by examples. The CSA-PD-EA model can provide the theoretical basis for chemical and material fields, such as the calculation of polymer physics and chemistry, the exploration of adsorption, assembly, activation and reaction of molecules, the multi-scale multicomponent complex electrochemical interface system, the multi-scale quantum dynamics theory, multi-scale structure and macro performance of complex systems. The performance and efficiency of the proposed model need to be further improved. In view of the shortcomings in modelling, we will further explore the deep integration of advanced artificial intelligence methods with dynamics in the future and promote the integrated development of physics, chemistry, materials and information disciplines.

## Data accessibility

The datasets supporting this article have been uploaded as part of the electronic supplementary material [60].
